# An Introduction to the *International Journal of Particle Therapy*'s Special Issue on Particle Therapy for Head and Neck Malignancies

**DOI:** 10.14338/2331-5180-8.1.1

**Published:** 2021-06-25

**Authors:** Steven J. Frank

Particle therapy has emerged as a standard-of-care treatment option in the management of head and neck malignancies owing to its unique physical and biologic properties that lead to improved clinical outcomes. Likewise, the value of particle therapy in the management of head and neck tumors has rapidly evolved over the past decade, and head and neck cancer management currently represents one of the most common indications for the use of proton therapy.

It is therefore my privilege to introduce this special *IJPT* Particle Therapy for Head and Neck Malignancies issue, which offers 33 articles by renowned international authors knowledgeable in head and neck cancer management, making it, to date, the most definitive guide to this new standard of care. The research presented herein expands our knowledge of the biologic enhancement effect of proton therapy, the physical properties that require unique and complex treatment planning approaches, and the resulting improvement in long-term clinical outcomes. Further clarity has been provided on the use of advanced Monte Carlo and LET-based treatment planning and quality assurance methods for emerging treatment centers, thereby minimizing the risk of unnecessary side effects and complications. Additional opportunities are offered for personalization of particle therapy through combining its biologic enhancement effects with concurrent systemic therapy. From pediatrics to adults, the issue seeks to advance health policy and improve patient access with model-based selection, activity-based costing, cost-effectiveness, financial toxicity, and work productivity outcomes.

I would like to dedicate this special issue to our head and neck cancer patients worldwide who have participated in the clinical trials and research protocols that have resulted in these publications, thereby advancing our knowledge and standard of care for future patients. I would also like to dedicate this special issue to my parents and family, who have provided constant support to see this project to completion.

We are most grateful for the efforts of our outstanding guest editors in this endeavor: Paul M. Busse, MD, PhD, from Massachusetts General Hospital; Lei Dong, PhD, from the Hospital of the University of Pennsylvania; Robert L. Foote, MD, from Mayo Clinic – Rochester; Piero Fossati, MD, MS, from MedAustron; Hans Langendijk, MD, PhD, from University Medical Centre Groningen; Nancy Y. Lee, MD, from Memorial Sloan Kettering Cancer Center; Jiade Lu, MD, MBA, from Shanghai Proton/Heavy Ion Center; Hsiao-Ming Lu, PhD, from the Hefei Ion Medical Center; Jun-Etsu Mizoe, MD, PhD, from Osaka Heavy Ion Therapy Center; Naruhiro Matsufuji, PhD, from the National Institutes for Quantum and Radiological Science and Technology National Institute of Radiological Sciences, Japan; Samir H. Patel, MD, of the Mayo Clinic – Phoenix; Juliette Thariat, MD, PhD, from Centre Baclesse / ARCHADE – Normandie Université; and Samir Patel, MD, of the Cross Cancer Institute and Stollery Children's Hospital, University of Alberta.

I also wish to thank *IJPT* Editor-in-Chief Dr. Nancy Mendenhall and Operating Editor Dr. Bill Mendenhall for their support of this important issue; Managing Editor Jessica Kirwan and Journal Manager Liza Winthrop Grammel, who worked tirelessly to see it to completion; Christine Wogan, for her substantive editing expertise; and Sasha F. Watt for her photography of the cover of the special edition.

**Figure:**
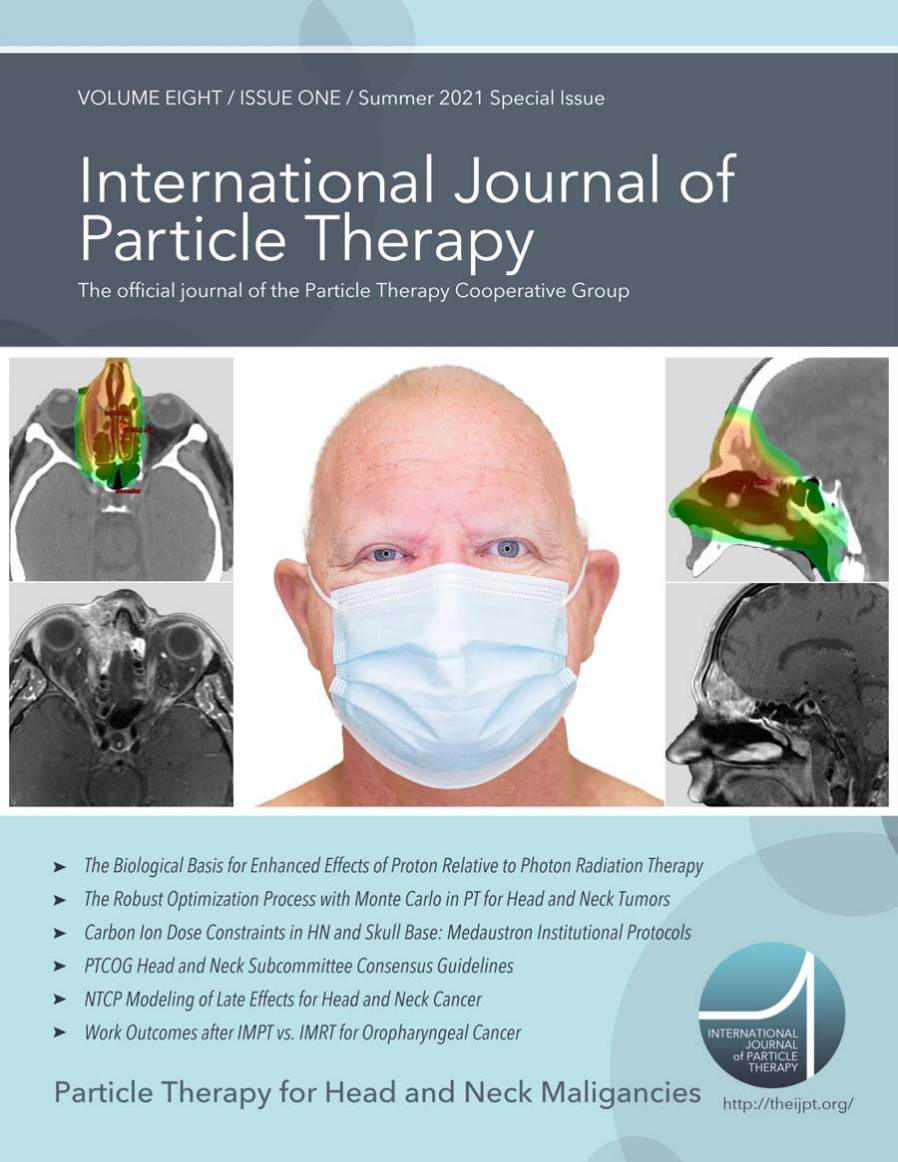


Finally, I would like to personally thank my gracious patient, Mr. Ronald L. Roberts, who kindly provided consent to use his image and name, and whose photograph and medical images are represented on the cover of this head and neck special issue. For tumors in this complex paranasal sinus area of the head and neck, proton therapy has become standard of care and is crucial to the pursuit of cure, cosmesis, and clear vision in this unique time of the COVID-19 pandemic.

